# Marrying Up by Marrying Down: Status Exchange between Social Origin and Education in the United States

**DOI:** 10.15195/v3.a44

**Published:** 2016-11-28

**Authors:** Christine R. Schwartz, Zhen Zeng, Yu Xie

**Affiliations:** a University of Wisconsin-Madison; b U.S. Department of Justice; c Princeton University and Peking University

**Keywords:** exchange marriage, intermarriage, homogamy, social stratification

## Abstract

Intermarriage plays a key role in stratification systems. Spousal resemblance reinforces social boundaries within and across generations, and the rules of intermarriage govern the ways that social mobility may occur. We examine intermarriage across social origin and education boundaries in the United States using data from the 1968–2013 Panel Study of Income Dynamics. Our evidence points to a pattern of status exchange—that is, persons with high education from modest backgrounds tend to marry those with lower education from more privileged backgrounds. Our study contributes to an active methodological debate by pinpointing the conditions under which the results pivot from evidence against exchange to evidence for exchange and advances theory by showing that the rules of exchange are more consistent with the notion of diminishing marginal utility than the more general theory of compensating differentials.

A common theme in theories of family change is that as societies modernize, parents lose control over their children’s choice of mates, and romantic love triumphs as the basis of partner selection (Goode 1963; Blau and Duncan 1967; Kalmijn 1991). Although the romantic love ideal is widespread in the United States (Buss et al. 2001), there is evidence that social background also plays an important role in mate selection. There are few studies of this topic, but those that do exist suggest a persistent tendency to match on social origin spanning from at least the 1940s through the 1980s (Blau and Duncan 1967; Burgess and Wallin 1943; Centers 1949; Kalmijn 1991; Charles, Hurst, and Killewald 2013). Furthermore, matching on social origin does not appear to be explained by the tendency for individuals with similar educational attainments to marry one another (Blau and Duncan 1967; Kalmijn 1991; Charles et al. 2013), suggesting that social background plays a direct role in mate selection and is not just a product of sorting on individuals’ own education.

In this article, we extend the literature on marital sorting by social background by examining the conditions under which departures from matching on social origin and education occur. Previous studies have focused on whether there is sorting on social origin over and above sorting on education but have not examined the relationship between the two forms of matching. We test the long-standing hypothesis that departures from spousal resemblance are characterized by *status exchange*—the notion that individuals compensate for the lack of one trait by offering other desirable traits to potential mates (e.g., Davis 1941; Merton 1941). In this context, status exchange marriages are those in which people with high social origins but low educational attainment marry those with low social origins but high educational attainment—in other words, people marry up in education by marrying down in social origin (and vice versa).

As Davis (1941) argues, intermarriage plays an important role in stratification systems. Whereas endogamy serves to reinforce social boundaries within and across generations, the rules of intermarriage govern the ways in which social mobility may occur. Popular accounts of socioeconomic status exchange often feature the social mobility of talented young men who marry women from wealthy backgrounds, a phenomenon captured by the phrase “marrying the boss’s daughter.” Such marriages may represent an exchange of wives’ future wealth inheritance for husbands’ current income stream or an exchange of inherited cultural and/or social capital for educational capital (Bourdieu 1984). From the perspective of social stratification and mobility, the exchange of social origin and education is particularly interesting because these two traits represent two dimensions of class standing—ascribed and achieved status. The relative importance of ascribed and achieved status in the allocation of life chances is seen as an indicator of the “openness” of a society’s stratification system (Hout 1988). Numerous studies have documented that achieved traits, education in particular, are increasingly important markers of social status as well as criteria of mate selection, whereas the roles of ascribed traits such as race, ethnicity, and religious background have weakened (e.g., Blau and Duncan 1967; Kalmijn 1991; Buss et al. 2001; Schwartz and Mare 2005; Qian and Lichter 2007; Rosenfeld 2008; Economic Policy Institute 2012: Figure 4N). In this article, we reexamine the role of social origin vis-à-vis education in mate selection by asking: to what extent do Americans leverage their class background to marry up in education?

Research on status-exchange marriage in the United States has often examined the exchange of socioeconomic status (SES) for racial status (e.g., Schoen and Wooldredge 1989; Kalmijn 1993; Qian 1997; Fu 2001; Gullickson and Torche 2014; Torche and Rich 2016). As part of a recent debate concerning whether or not race–SES exchange exists in the United States, Kalmijn (2010:1262) proposed that research on exchange be expanded to other contexts: specifically, to different ethnic groups or countries. Another way of extending the literature is to examine how generalizable exchange is to other characteristics (for example, social background and educational attainment). Indeed, while a large fraction of research on exchange has focused on race–SES exchange, a major focus of Davis’s seminal work on intermarriage was the exchange of social background for other desirable traits such as “achievement, beauty, intelligence, youth, [and] wealth” (1941:386). Consistent with the exchange of social background for education, Blackwell (1998) found that parental education improves women’s chances of marrying highly educated men net of women’s own education, but she did not directly test whether individuals trade their relative advantage on one trait for their spouses’ relative advantage on another. Rather, she conceptualized parental education as a resource allowing individuals to obtain a better match on a single characteristic (spouses’ education). Thus, to our knowledge, this is the first empirical test of the exchange hypothesis with respect to social origin and education in the United States or elsewhere.

In light of the debate on the appropriateness of log-linear analyses in the literature on status exchange (see Rosenfeld 2005; Gullickson and Fu 2010; Kalmijn 2010), we also review and synthesize the methods that have been used to evaluate the exchange hypothesis. Our methodological discussion focuses on methods of separating exchange from other social forces that influence mate selection (such as a tendency for men or women to choose partners similar to themselves) and is part of continuing efforts to clarify the important methodological and substantive implications of this debate (Gullickson and Torche 2014). We apply a “step-by-step” approach to the study of exchange, adding controls one by one to identify the most important confounders. Previous research has debated the value of various log-linear models to identify exchange but has not systematically identified why different specifications yield different results. We demonstrate that models that control for the correlation between education and social origin within individuals consistently yield positive evidence on exchange; on the other hand, simple tests that do not control for this correlation consistently reject the exchange hypothesis.

Finally, we advance exchange theory by refining the usual version of the exchange hypothesis, which is based on the notion of *compensating differentials*, with a more specific hypothesis based on *diminishing marginal utility*. We show that observed matching patterns support the more specific version of exchange based on the notion of diminishing marginal utility.

## Potential Mechanisms of Exchange Marriage

### Compensating Differentials

Current theories of mate selection are primarily based on the idea of the “marriage market” (Becker 1973). Within this framework, marriage is seen as an exchange of resources between partners, and marital selection follows the principle that each person attempts to find a suitable mate subject to market constraints. The sociological literature distinguishes two types of preferences in mate selection: (1) people may exhibit vertical preferences and seek highly ranked mates and (2) they may show horizontal preferences and seek in-group mates. Spousal resemblance on a trait—referred to as homogamy—occurs either because people simply prefer partners who are similar to themselves or because they are competing for the most highly ranked partner (Schwartz 2013; Xie, Cheng, and Zhou 2015). As men and women attempt to marry up and reject suitors of lower rank than themselves, marriages will tend to be contracted between partners with similar characteristics.

Status exchange is a specific pattern of intermarriage involving two (or more) hierarchical traits—one partner has a relative advantage in one trait but a relative disadvantage in the other. Why do partners exchange traits? A general explanation of exchange, commonly found in the literature, is *compensating differentials* (e.g., Gullickson and Fu 2010; Kalmijn 2010; Rosenfeld 2005). Just as workers need to be paid more to take a hazardous job, in a competitive marriage market people pay a “price” to marry those with a desirable trait they do not have themselves (Grossbard-Shechtman 1993).

As the number of characteristics that individuals sort on increases, it becomes increasingly difficult to form a perfectly homogamous match (Cheng and Xie 2013). Status exchange can be seen as a special type of intermarriage across group boundaries or, rather, a complex form of homogamy, in which marriages are based on a balance of pluses and minuses on two or more traits—or overall “trade values.” Because mate selection is based on multiple traits, a lack of one desirable trait can be compensated for by an advantage in another trait. Competition for the most desirable mate will thus result in a pattern of matching on individuals’ overall trade values rather than strict matching on individual traits. The notion of compensating differentials extends to more than two traits; the important point is that the sum of the pluses and minuses balance one another within couples. In this study, we test the following formulation of the exchange hypothesis with respect to education and social origins.

Hypothesis 1: Among couples who intermarry across social origin and education groups, the partner with higher education will tend to have lower social origins and the partner with lower education will tend to have higher social origins.

Note that the notion of compensating differentials only predicts that the sums of the pluses and minuses across traits are roughly equal between spouses; it does not predict a particular pattern or direction to the balancing of traits. To illustrate this, consider a marriage market in which all men and women are scored on a scale of 1 to 10 for their education and social origin, and mate selection is based purely on those scores. Also, assume that everyone has the same preferences for education and social origin. Let us examine the choice of a man with an education score of 6 and a social origin score of 4 (E6O4 for short). The mechanism of compensating differentials predicts that if this man marries across boundaries, he is more likely to marry a woman of equal marriageability—that is, someone with the same sum score of 10 (e.g., E7O3 or E4O6)—than a woman of higher or lower ranks in both traits (e.g., E7O5 or E5O3). Among the various types of women with a sum score of 10, this mechanism does not indicate which type he prefers. For example, the man might marry a woman of E7O3 or he might marry a woman of E4O6. Both of these marriages are exchange marriages because in each the partner with relatively higher educational attainment has relatively lower social origins, but they differ in terms of who has the advantage on which trait.

### Diminishing Marginal Utility

We further hypothesize that patterns of exchange are consistent with the notion of diminishing marginal utility. The economic law of diminishing marginal utility states that as a person increases consumption of one good (while keeping consumption of other goods constant), there is a decline in the utility the person derives from consuming an additional unit of that good. Applied to intermarriage, diminishing marginal utility predicts that people with unequal endowments (e.g., those who are highly educated but come from humble social origins) may trade the trait they have in abundance (e.g., their education) for the trait they lack (e.g., their social origins). This leads to the following specific pattern of exchange.

Hypothesis 2: People who are highly educated relative to their social origins tend to form exchange marriages (exchanging education for social origins) with those who have higher social origins relative to their educational attainment.

In contrast to the notion of compensating differentials, which proposes a general balancing rule for intermarriages, the mechanism of diminishing marginal utility predicts the direction of exchange based on relative endowment. This difference allows us to empirically adjudicate between the specific and the general patterns of exchange. Following the example above, by the general rule of compensating differentials, E4O6 and E703 are equally preferable partners to a man with an endowment of E6O4, but the law of diminishing marginal utility predicts that E6O4 prefers E4O6 to E703. In forming exchange marriage with the former, he trades the trait he has in abundance (education) for the trait he lacks (social origins). This brings him more utility than marrying the latter, where he exchanges the trait he lacks (social origins) for the trait he already has in abundance (education). It follows that if the law of diminishing marginal utility is at work, exchange marriage is most likely formed between a man and a woman with complementary traits; exchange marriage is less likely between men and women looking to trade up in the same trait. On the other hand, if only the rule of compensating differentials is at work, then the pattern of exchange should not vary substantially by couple’s own endowment. It should be noted that evidence for the hypothesis of diminishing marginal utility does not invalidate the hypothesis of compensating differentials. Rather, it is a refinement of the more general theory.

### Gender Differences in Exchange

Another explanation for exchange marriage is that different traits are valued in male and female partners because of gendered roles in family and work lives (Buss and Barnes 1986). By exchanging traits, individuals can match with partners who have more qualities they value than by finding their exact match. The conventional view in stratification research has been that husbands’ schooling is more closely related to family income and class identification than wives’ schooling (Sørensen 1994), although this may change as women’s labor force participation increases. Therefore, if men and women attempt to maximize family socioeconomic standing in choosing partners, we might expect the exchange of wives’ social origins for husbands’ education to be more common (“marrying the boss’s daughter”) than the other way around (“marrying the boss’s son”).

If, on the other hand, education and social origins are equally valued in men and women—as markers of life styles, cultural values, and attitudes (Bruze 2011; Kalmijn 1994)—then we might expect a gender symmetric exchange pattern or no exchange at all. Indeed, the only previous research to our knowledge that has tested whether women are more likely to “marry up” in social background than men found no gender difference in the 1960s (Rubin 1968).

To test gender differences in exchange, we estimate the exchange of husband’s education for wife’s social origins and the exchange of husband’s social origins for wife’s education separately and test the significance of this difference in various analyses.

## Data and Measures

Our analysis uses data from the 1968–2013 Panel Study of Income Dynamics (PSID), a longitudinal study that originated in 1968 with a nationally representative sample of households and an oversample of low-income households. An issue with the PSID is that while the sample was representative of the population in 1968, the nature of the U.S. population has changed substantially since the late 1960s because of immigration. However, previous studies have shown that when weights are used, it produces estimates of marriage formation that are consistent with findings from other sources (Lillard and Panis 1998).

To minimize selection bias because of divorce, we examine new marriages formed within five years of the time they were first observed in the survey. Because of the prospective nature of the survey, the vast majority of marriages in our sample were first observed very close to the time they were formed; 90 percent of marriages in the sample were formed within two years of the interview. We do not include the Latino oversamples available in the PSID, as these families were only interviewed from 1990–1995 (Gouskova et al. 2008). To maximize the data available for analysis, we include first and later marriages. Sensitivity tests indicate that our results are robust to the exclusion of remarriages. The total sample size with complete information on both spouses’ education and social origin is 7,398.^1^

For both husbands and wives, education is measured as years of schooling and coded as a categorical variable with seven levels: 1–5, 6–8, 9–11, 12, 13–15, 16, and 17+. Social origin is defined as parental socioeconomic status and operationalized as the sum of the years of schooling of both parents. It is also collapsed into seven categories: 6–14, 15–17, 18–21, 22–23, 24, 25–28, and 30–36, each of roughly equal proportions.

We chose parental education rather than parental income or occupation as the measure of social origin for several reasons. First, education is more stable than income or occupation. Second, because education is a good measure of status for both men and women, it allows us to account for the socioeconomic status of both parents. In comparison, income or occupational standing does not capture the mother’s status well because a high percentage of mothers in the early years of the PSID are homemakers. Third, the PSID measured parental education in most years, whereas parental income and occupation are available for a smaller subset of waves. Thus, using parental income or occupation as the measure of social origin would result in a significant reduction of sample size. We therefore focus on parental education as our measure of social origin.

Given our focus on the effects of various controls on the test of exchange, we chose simple measures of education and social origins in presenting our main results. To ensure that our results are not affected by our choice of measure, we conducted sensitivity analyses using alternative measures (e.g., father’s occupational standing). We also repeated our analysis with alternative categorizations of education and social origin and methods for handling the contribution of mothers’ and fathers’ education. The results of these sensitivity tests are reported following the main results below.

## Testing the Exchange Hypothesis: A Step-By-Step Approach

Since the race–education exchange hypothesis was outlined more than 70 years ago by Merton (1941) and Davis (1941), many studies have attempted to verify or refute it. However, only recently have researchers begun to scrutinize the methods used to test the hypothesis. In a critique of the literature, Rosenfeld (2005) distinguished between “simple” and “complex” approaches to testing the exchange hypothesis. As he observed, early studies relying on cross-tabulations to assess whether interracial marriages tended to be formed by whites with low education marrying highly educated blacks generally rejected the exchange hypothesis (Bernard 1966; Heer 1974; Monahan 1976). By contrast, recent studies using “complex” log-linear models have often supported the hypothesis (Schoen and Wooldredge 1989; Kalmijn 1993; Qian 1997; Fu 2001; Gullickson 2006; Gullickson and Torche 2014; Torche and Rich 2016). Rosenfeld’s own inclination was to use simple methods because the results of complex models depend on model assumptions, whereas “[s]imple tabular analyses at least have the advantage of transparency” (2005:1287). However, he did not explain why “simple” and “complex” approaches tend to lead to opposite conclusions.

In their responses to Rosenfeld (2005), Gullickson and Fu (2010) and Kalmijn (2010) argue for the “complex” log-linear modeling approach but, like Rosenfeld, did not systematically explore why the “simple” and the “complex” methods yield different results. We address this puzzle by applying a “step-by-step” approach to the study of intermarriage by social origin and education. Beginning with the simplest test of exchange, we add controls one by one using a series of nested log-linear models and compare the results across these models. Through this approach, we identify the most important confounder of exchange: the intertrait correlation within individuals (i.e., highly educated people tend to come from higher social origins). Because log-linear models typically control for intertrait correlations while simple methods do not, this explains why the two methods tend to yield opposite results. This demonstration will be useful to analysts who face the task of choosing a conceptually defensible model to test exchange theory. While we focus on education and social origin here, the methodological discussion below applies to the exchange of any two traits such as race and SES or attractiveness and education.

Table 1 classifies marriages by spouse’s relative education (E) and social origin (O). We denote the proportion of couples in the *i*th row and the *j*th column by *P*[*i, j*], with (*i* = 1, 2, 3, *j* = 1, 2, 3). Tests of exchange involve at least four types of marriages, as shown in Table 1: (1) marriages in which the husband exchanges his higher education for his wife’s higher social origin (cell [3,1]); (2) marriages in which the wife exchanges her higher education for her husband’s higher social origin (cell [1,3]); (3) marriages in which both of the wife’s traits are higher than her husband’s (cell [1,1], which we refer to as “wife-advantaged” marriages); and (4) marriages in which both of the husband’s traits are higher than his wife’s (cell [3,3], which we refer to as “husband-advantaged” marriages). Marriages in the other cells of Table 1 are homogamous with respect to education and/or origin. These cells serve as reference points in the more complex tests of the exchange hypothesis.

As Table 2 shows, newly married women between 1968 and 2013 were about equally likely to “marry up” as “marry down” with respect to education in the United States (28.1 percent vs. 27.9 percent),^2^ while it was somewhat more common for men to “marry up” in social origin than women (38 percent vs. 32 percent). Most marriages (62 percent) are homogamous on one or the other trait, while only 14 percent of all marriages are homogamous on both traits, demonstrating the potential difficulty of finding a match across multiple traits. The exchange of husband’s education for wife’s social origin is more common than the other way around (9 percent vs. 7 percent), consistent with the stereotype of the young upstart man “marrying the boss’s daughter.” Together, exchange marriages account for about 15 percent of all marriages.

### 1. The Simple One-Group Test

A simple test of exchange can be written as:

(1a) Exchange of husband’s E for wife’s O:P[3,1]/P[1,1]>1

(1b) Exchange of wife’s E for husband’s O:P[3,1]/P[3,3]>1

where *P*[*i, j*] is the proportion of couples in cell [*i, j*] in Table 1. The numerators *P*[3,1] and *P*[1,3] are exchange marriages, and the denominators *P*[1,1] and *P*[3,3] are marriages in which one partner has a relative advantage in both traits. Equations (1a) and (1b) provide a simple test of exchange by comparing the observed frequencies of exchange marriages to those of wife-advantaged or husband-advantaged marriages among couples who have different social origins. This test is conceptually equivalent to comparing the frequency of mixed-race marriages in which the black spouse has more education (exchange) to that in which the white spouse has more education (that is, the white spouse has both higher educational and racial status) (e.g., Tables 1 and 2 and Figure 3 of Rosenfeld 2005). We refer to this test as the *simple one-group test* because it examines mixed-origin (or mixed-race) couples only, without using a comparison group such as same-origin (same-race) couples.

As Tables 2 and 3 show, the simple one-group test yields a *P*[3,1] / *P*[1,1] ratio of 0.085 / 0.127 ≈ 0.66 < 1 and a *P*[1,3] / *P*[3,3] ratio of 0.069 / 0.104 ≈ 0.66 < 1 (differences between the ratios calculated from Table 2 and those shown in Table 3 are due to rounding), ratios that are inconsistent with the exchange of husbands’ education for wives’ origin and wives’ education for husbands’ origin, respectively.

However, the one-group test may be confounded by educational hypergamy or hypogamy. Hypergamy (hypogamy) is a general tendency for women to “marry up (down)” on a trait. If educational hypergamy predominates, there may be more couples in which husbands have more education than their wives but have lower social origins (*P*[3,1]) than couples in which husbands have both less education and lower social origins than their wives (*P*[1,1]), merely because of a tendency for women to “marry up” in education rather than because men and women are exchanging social origin and education. Conversely, a low ratio of *P*[1,3] / *P*[3,3] may indicate a male aversion to marrying women of higher education than themselves rather than the absence of exchange.

### 2. The Two-Group Test Controlling for Hypergamy

To control for an overall tendency toward educational hypergamy or hypogamy, we can use couples with the same social origins (column 2 in Table 1) as reference groups. This converts the simple one-group test in Equations (1a) and (1b) to a two-group test shown in Equations (2a) and (2b):

(2a) $$\text{Exchange of HE for WO}:\frac{P[3,1]/P[1,1]}{P[3,2]/P[1,2]}>1$$

(2b) $$\text{Exchange of WE for HO}:\frac{P[1,3]/P[3,3]}{P[1,2]/P[3,2]}>1$$

In the numerator of (2a) is the simple one-group test (1a) for the exchange of husbands’ education for wives’ social origin. The denominator of (2a) expresses the tendency for educational hypergamy—that is, the likelihood that husbands have more education than their wives relative to the likelihood that wives have more education—among couples who share the same social backgrounds. Equation (2b) is the analogous test for the exchange of wives’ education for husbands’ social origin. Equations (2a) and (2b) use the tendency for educational hypergamy among origin-homogamous couples as controls when estimating educational hypergamy among exchange couples. In other words, Equation (2a) and (2b) estimate exchange over and above a general tendency for hypergamy or hypogamy.^3^ The idea here is analogous to the difference-in-difference method in econometrics. We refer to the cross-product (ratio-of-ratio) in Equations (2a) and (2b) as *the odds ratio measure of exchange marriage*. Higher odds ratios indicate a greater likelihood of exchange marriage, controlling for educational hypergamy.

As Tables 2 and 3 show, the odds ratio for the exchange of men’s education for women’s social origin from Equation (2a) is (0.085 / 0.127) / (0.082 / 0.083) ≈ 0.68 and that for the exchange of women’s education for men’s social origin from Equation (2b) is (0.069 / 0.104) / (0.083 / 0.082) ≈ 0.65. Thus, like the one-group test, the simple two-group test is also inconsistent with the exchange hypothesis. The numerical results of this test are similar to those of the one-group test, suggesting that controlling for educational hypergamy has almost no effect on the test of exchange in our case. This is not surprising given that there was only a very weak tendency for women to marry up in education over this period, as we saw from column 2 as well as the row marginals of Table 2.

Previous studies using the two-group test controlling for hypergamy (e.g., Bernard 1966; Liang and Ito 1999; Fu 2008) have also rejected the race–education exchange hypothesis. As pointed out by several researchers (e.g., Heer 1974; Kalmijn 2010), however, this test tends to be biased against the exchange hypothesis by failing to account for the association between race and education. Next, we explain how an association between traits could bias the test of exchange and outline a correction for that bias.

### 3. The Two-Group Test Controlling for Hypergamy and the Joint Distribution of Correlated Traits

Social origin and education are positively correlated within individuals: people of higher social origin often have higher education themselves. So, if men and women were randomly paired, the partner of higher social origin would also tend to be the one with more education. This pattern is just the opposite of exchange marriage, in which each partner offers a relative advantage in one trait to compensate for a relative disadvantage in another trait. In other words, given the positive association between education and social origin, we would expect to see more husband- or wife-advantaged marriages than exchange marriages simply by chance. Tests 1 and 2 do not control for this and as a result may incorrectly reject the exchange hypothesis.

Referring back to Equations (2a) and (2b) for the two-group test, one can see why the intertrait correlations would stack the decks against exchange. Given that people with more education tend to come from privileged social backgrounds and that people with less education tend to come from modest backgrounds, the most common pairings one would expect under random matching would be wife-advantaged and husband-advantaged pairings (*P*[1,1] and *P*[3,3]). In contrast, exchange marriages involve partners that are generally more rare in the population, namely, those that are higher on either education or social origin but lower on the other trait. Because the numerators of Equations (2a) and (2b) are divided by the proportions of wife-advantaged and husband-advantaged couples that are biased upward because of the correlation between education and social origin, the two-group test will be biased against exchange. The same bias exists for the one-group test.

To control for the abundance of wife-advantaged and husband-advantaged couples that would occur through chance alone because of the positive correlation between education and social origin, test 3 compares the observed odds ratio of exchange (Eqs. [2a] and [2b]) to what would be expected by chance given the distribution of husbands’ and wives’ education and social origin and the tendency for educational hypergamy or hypogamy. This test can be represented as:

(3a) $$\text{Exchange of HE for WO}:\frac{P[3,1]/P[1,1]}{P[3,2]/P[1,2]}/\frac{p[3,1]/p[1,1]}{p[3,2]/p[1,2]}>1$$

(3b) $$\text{Exchange of WE for HO}:\frac{P[1,3]/P[3,3]}{P[1,2]/P[3,2]}/\frac{p[1,3]/p[3,3]}{p[1,2]/p[3,2]}>1$$

where *p*[*i*, *j*] refers to the expected proportion in cell [*i*, *j*] under random matching given the observed distribution of spouses’ traits and the observed within-person association between social origin and education. The exchange hypothesis is supported if the observed odds ratio exceeds the expected odds ratio. Controlling for the joint distribution of correlated traits generally works in favor of the exchange hypothesis, as the expected odds ratios of exchange marriages under random matching are smaller than 1. This can be derived by noting that, given the positive correlation between education and social origin, the expected relative risk of educational hypergamy versus hypogamy increases with husband’s relative status in social origin: *p*[3,1] / *p*[1,1] < *p*[3,2] / *p*[1,2] < *p*[3,3] / *p*[1,3]. Because many traits that have been studied in the context of exchange marriage are positively correlated (e.g., racial status and socioeconomic status), failing to control for intertrait correlations generally biases against the hypothesis of exchange. For the case of race–education exchange, Heer (1974) demonstrated that controlling for racial differences in education in this way greatly improves the predictive power of the exchange theory. This is also true for the exchange of education and social origin, as we will demonstrate.

Simple tabular analyses do not control for husbands’ and wives’ characteristics, as the calculation of the expected odds ratios of spousal differences is algebraically complicated in four-way marriage tables (HE × HO × WE × WO). However, controlling for the distribution of husbands’ and wives’ characteristics is standard practice with log-linear models. Indeed, log-linear analysis is a popular method in studies of assortative mating because of this feature. To control for the joint distribution of two traits, researchers can include dummy variables for both traits of husbands and wives (i.e., all four one-way marginal distributions HE, HO, WE, and WO) as well as dummies for the within-person intertrait associations (i.e., HE × HO and WE × WO).^4^ The expected odds ratios of exchange under random matching can be calculated from the predicted frequencies of marriage with a simple log-linear model with dummy variables for HO, HE, WO, WE, HE × HO, and WE × WO only.

**Figure 1:**
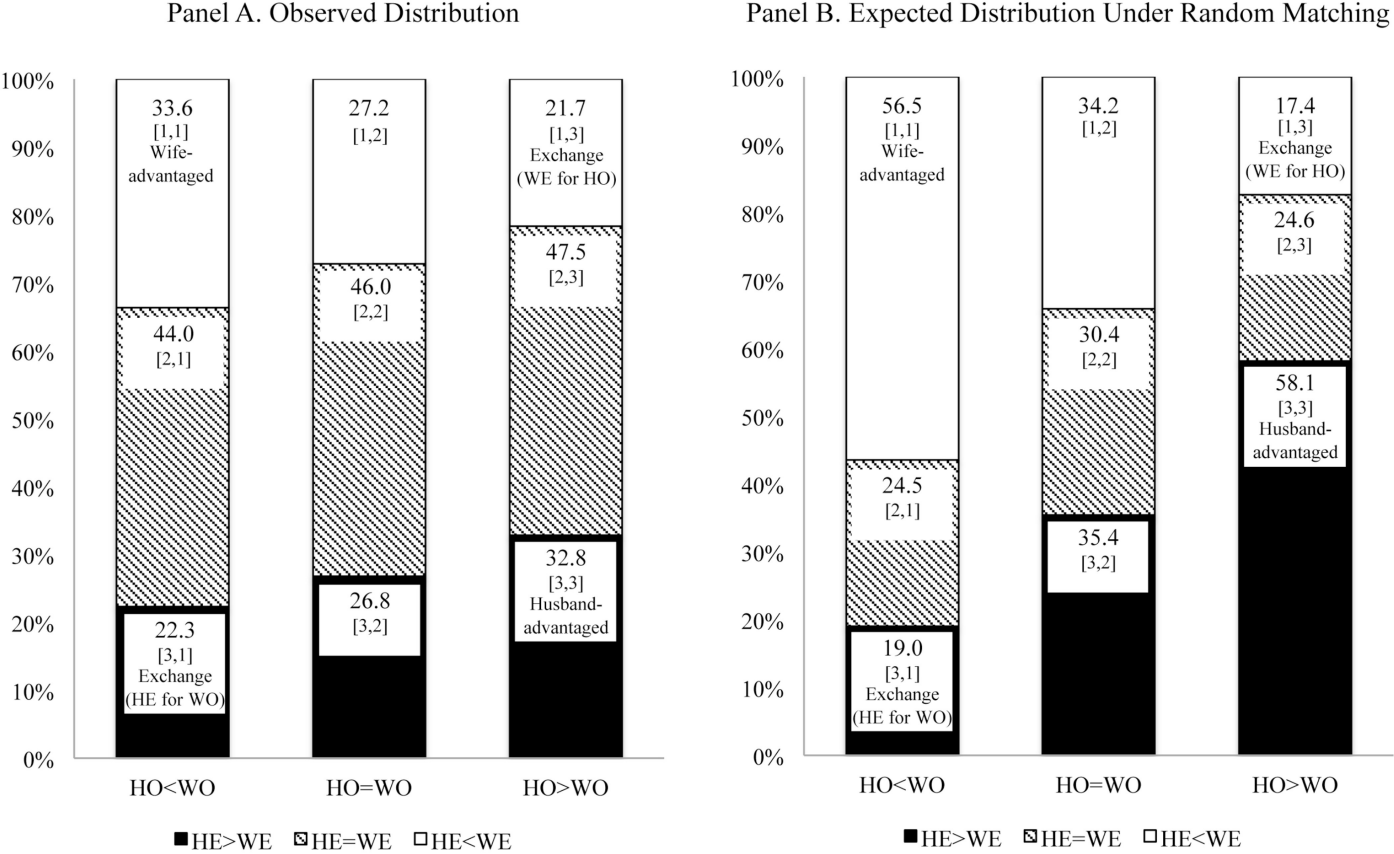
Spouse’s relative education by relative social origin. Notes: HE = husband’s education; WE = wife’s education; HO = husband’s social origin; WO = wife’s social origin; hypogamy = wives "marry down"; homogamy = spouses share traits; hypergamy = husbands "marry down. " Cell row (i) and column (j) positions of Table 1 are shown in brackets [i, j]. Total sample size n = 7,398.

Comparing test 3 to test 2 in Table 3 shows that evidence for exchange emerges after controlling for the within-person association between education and social origins (the odds ratios of exchange are >1) and the tests are both significant at *p* < 0.01. Figure 1 explains how this happens. Panel A shows that there are more husband- or wife-advantaged marriages than exchange marriages in our data. But panel B shows that under random matching there would be even more husband- or wife-advantaged marriages and fewer exchange marriages. This suggests that men and women are systematically avoiding husband- or wife-advantaged configurations and selecting exchange marriages.

We can use the column percentages in Figure 1 to compute Equation (3a), which gives the same result as using the cell percentages. This gives [(22.3 / 33.6) / (26.8 / 27.2)] / [(19.0 / 56.6) / (35.4 / 34.2)] ≈ 2.07 (see Table 3), evidence that husbands exchange their education for wives’ social origins. Evidence that wives exchange their education for husbands’ social origins can be obtained in the same way.

In sum, many husband- and wife-advantaged marriages are the result of the well-known association between social origin and education for both spouses. Test 3 removes this confounding factor by comparing the observed proportion of husband- and wife-advantaged marriages to the expected proportion of such marriages under random matching. Test 3 yields strong evidence for exchange, reversing the direction of previous tests. Controlling for the joint distribution of correlated traits is the key factor that explains differences between the “simple” and “complex” tests as we further demonstrate below.

### 4. The Two-Group Test Controlling for Hypergamy, the Joint Distribution of Correlated Traits, and Homogamy

Test 3 is limited in that the expected odds ratios of exchange are calculated under the naive assumption of random matching. Note that the observed percentages of educational homogamy in panel A of Figure 1 are significantly higher than the expected percentages in panel B regardless of couples’ relative origin status, indicating a strong tendency to match on education. The tendency to choose mates of similar standing is well established, and the hypothesis of status exchange has always been about whether exchange across two or more traits operates in the marriage market, net of the strong tendency toward homogamy within any given trait (Rosenfeld 2005). This requires that the test of exchange further control for homogamy. Hence, we have:

(4a) $$\text{Exchange of HE for WO}:\frac{P[3,1]/P[1,1]}{P[3,2]/P[1,2]}/\frac{p\prime [3,1]/p\prime [1,1]}{p\prime [3,2]/p\prime [1,2]}$$

(4b) $$\text{Exchange of WE for HO}:\frac{P[1,3]/P[3,3]}{P[1,2]/P[3,2]}/\frac{p\prime [1,3]/p\prime [3,3]}{p\prime [1,2]/p\prime [3,2]}$$

where *p*′[*i*, *j*] refers to the expected proportion in the [*i*, *j*] cell under a model that accounts for the joint distribution of correlated traits and the tendency for homogamy on education and social origin.

Past studies using log-linear models to test the exchange hypothesis have typically controlled for racial and educational homogamy in addition to the distribution of correlated traits and thus generally fall under the category of test 4. There are two ways to implement the test. Kalmijn (1993) first estimated a model controlling for the joint distribution of correlated traits and homogamy and then compared the observed and estimated “hypergamy ratios”—which he defined as the ratio of educational hypergamy to that of educational hypogamy—for mixed-race and same-race couples.^5^ Tests 3 and 4, as outlined above, use the same two-step approach. Some researchers (e.g., Gullickson 2006; Rosenfeld 2005) estimated “exchange parameters” that distinguish exchange marriages from wife- or husband-advantaged marriages directly using log-linear models while controlling for the above-mentioned confounders. The two approaches are conceptually similar (although the numerical results may differ slightly), but it is much easier to perform statistical tests with the latter, one-step approach (see also Gullickson 2006 and Gullickson and Torche 2014). Below, we present our formal tests of exchange using the one-step approach.

As noted by Rosenfeld (2005), the test of exchange may be sensitive to how we control for homogamy. Hence, we test exchange under a series of log-linear models with various specifications for homogamy in education and social origin. Our analysis reveals that it is the within-person correlation between education and social origin—rather than homogamy or hypergamy—that is mainly responsible for the contradictory results of the log-linear analyses and simple analyses. Our models take the following form:

$$\text{log}(E[F_{ijkl}])=HE_{i}+HO_{j}+WE_{k}+WO_{l}+{\left[HE\times HO\right]}_{ij}+{\left[WE\times WO\right]}_{kl}\;+{\left[HE\times WE\right]}_{ik}+{\left[HO\times WO\right]}_{jl}+D_{HE>WE}+D_{HO>WO}+D_{HE>WE,HO>WO}\;+D_{HE>WE,HO<WO}+D_{HE=WE,HO>WO}+D_{HE=WE,HO<WO}$$

The dependent variable *E*[*Fijkl*] is the expected frequency of marriages between men of the *i*th educational category and the *j*th social origin category and women of the *k*th educational category and the *l*th social origin category. The parameters are interpreted as follows.

1. HE, HO, WE, and WO control for the marginal distributions of men and women in categories of educational attainment and social origin. HE × HO and WE × WO control for the associations of education and social origin among men and women, respectively. Together, these parameters adjust for the joint distributions of correlated traits, while the rest of the parameters characterize how matching patterns deviate from random matching.
2. HE × WE and HO × WO capture patterns of homogamy in education and social origin. We model homogamy in six different ways to see if our test of exchange is robust. In each model, we use the same pattern for educational homogamy (HE × WE) and origin homogamy (HO × WO). The six homogamy patterns are described in Table 3 and fit statistics for these models are shown in the online supplement.
3. D_HE>WE_ and D_HO>WO_ are dummy variables controlling for the general female tendency to marry up (or down) in status with respect to education and social origin.
4. D_HE>WE,HO<WO_, D_HE<WE,HO>WO_, D_HE=WE,HO>WO_, and D_HE=WE,HO<WO_ are dummy variables capturing the interactions of spouses’ relative statuses in education and social origin. There are two exchange parameters: D_HE>WE,HO<WO_ (= 1 if HE>WE & HO<WO and = 0 if otherwise), which captures the exchange of HE for WO, and D_HE<WE,HO>WO_ (= 1 if HE<WE & HO>WO and = 0 if otherwise), which captures and the exchange of WE for HO. Husband- and wife-advantaged marriages are treated as the reference groups. Thus, in exponential form, the exchange parameters are interpreted as the ratio of exchange marriages to husband- or wife-advantaged marriages, controlling for correlated traits, homogamy, and hypergamy.

As Table 3 shows, the exponentiated exchange parameters range from 1.34 to 2.38 for the exchange of HE for WO and from 1.41 to 2.64 for the exchange of WE for HO. All parameters are statistically significant, with *p*-values below 0.001. This means that depending on the model specification, the likelihood of exchange marriage exceeds that of husband- or wife-advantaged marriages by 34 percent to more than 160 percent after controlling for the joint distribution of correlated traits and tendencies for hypergamy and homogamy. Because a higher ratio indicates a stronger pattern of exchange, the results suggest that exchange is more pronounced in models 1 and 2, which have simpler controls of homogamy. As distance parameters are introduced to better control for homogamy in model 3, the statistical pattern of exchange becomes weaker but is still highly significant. Overall, the results from log-linear models indicate that the test of exchange is not sensitive to how we model homogamy. Rather, a persistent pattern of exchange is found as long as we control for the distribution of correlated traits.

## Testing Gender Differences in Exchange

The exchange parameters are similar for men and women in all six models. None of the differences by sex (exchange of HE for WO versus WE for HO) within models are statistically significant. Thus, we are unable to reject the null hypothesis that the exchange of education and social origin is symmetric by sex.

The conventional view in stratification research has been that husbands’ socioeconomic characteristics are more closely related to family class identification than wives’ socioeconomic characteristics (Sørensen 1994), and therefore if men and women attempt to maximize family socioeconomic standing in choosing partners, we might expect the exchange of wives’ social origins for husbands’ education to be more common (“marrying the boss’s daughter”) than the other way around (“marrying the boss’s son”). But if social origins and education are also markers of life styles, cultural values, and attitudes in the marriage market (Bruze 2011; Kalmijn 1994), then we might expect a symmetric association. Indeed, the only article to our knowledge that has tested whether women are more likely to “marry up” in social background than men also found a relatively symmetric association (Rubin 1968).

It is possible that “marrying the boss’s daughter” was more common in the past than at present. However, we tested for period effects in our data and found no evidence of such a trend from 1968–1979 to 1980–1991 and 1992–2013 or evidence of gender difference in exchange within any of those time periods. There is robust evidence for exchange in each of these periods.

## Exchange Marriage and Diminishing Marginal Utility

Thus far, our results are consistent with the general notion of compensating differentials (hypothesis 1)—that is, men and women tend to form matches that balance across their pluses and minuses on the marriage market in the absence of homogamy. Next, we test hypothesis 2, based upon the notion of the diminishing marginal utility, the idea that people with a relative advantage in education tend to form exchange marriages with partners who have a relative advantage in social origin.

We define relative advantage in this context as the sign of the difference between a person’s education score and social origin score. Marriages are grouped by couple’s configuration of relative advantages (see Table 4). (A1) is the subsample of couples in which husbands’ education exceeds their social origins and wives’ social origins are equal to or greater than their education (HE>HO & WE≤WO). (B1) is the subsample of couples in which wives’ education exceeds their social origins and husbands’ social origins are equal to or greater than their education (WE>WO & HE≤HO). Subsamples (A1) and (B1) are those for which we expect the likelihood of exchange to be greatest given that husbands and wives will trade traits they have in abundance for those that they have relatively little of. We expect exchange to be less common among other couples. We compare the likelihood of exchange in these subsamples to the likelihood of exchange for other couples who either both have the same or higher social origins than education (HE≤HO and WE≤WO) or both have higher education than social origins (HE>HO and WE>WO). To test the hypothesis that exchange is more likely in subsamples (A1) and (B1), we interact the exchange parameters with dummy variables for couples’ relative advantage. The model also includes HE, HO, WE, WO, HE × WE and HO × WO as well as distance parameters and variable homogamy controls, as in model 4 of Table 3, the preferred model by Akaike’s (1973) information criteria (see the online supplement).^6^

The results are presented in Table 4. The exponentiated exchange parameters displayed here have the same interpretation as those in Table 3. They are ratios of the proportion of exchange marriages to that of wife- or husband-advantaged marriages—controlling for hypergamy, homogamy (as in model 4), and the joint distribution of correlated traits—but are estimated separately by couples’ relative advantage. Consistent with hypothesis 2, the exchange parameters are only statistically significant when spouses trade a trait that they have in abundance for a trait they have relatively little of. Exchange for other couples is less common, although the difference in magnitude is statistically significant only for the exchange of wife’s education for husband’s social origins (*p*-value = 0.016). Taken as a whole, our results suggest that exchange depends on couples’ relative endowments. Put differently, exchange is directed in that people are more likely to exchange what they have in abundance for what they lack rather than the other way around.

## Sensitivity Tests

### Measure of Social Origins

Our measure of social origins is based on mother’s and father’s educational attainments, but it is possible that the results would vary if alternate measures were used. To test this, we replicated our analyses with an alternative measure of social origin for husbands—father’s occupational standing—operationalized as an ordinal variable with seven categories ranked by mean occupational education.^7^ Because parental occupation is not consistently available for anyone other than husbands’ fathers, our alterative measure of social standing for husbands is father’s occupational standing (without reference to mother’s occupational status or education), and wives’ social origins are measured as in the analyses above (the sum of mother’s and father’s education). We use data from 1968 to 1993 for this test, the period for which the measure of father’s occupation is consistent in the survey. The results show weaker odds ratios of exchange marriage, which is likely due to the asymmetric measurement of parental social standing, but the main findings presented above persist.

Operationalizing social origins as the sum of parents’ years of schooling as we have done in our main analysis assumes that both parents’ statuses have equal weight on the marriage market. To test the sensitivity of our results to this assumption, we estimated our models first using only father’s educational attainment as our measure of social origins and then using only mother’s educational attainment. Again, our conclusions are not affected.

Another complication is that family lives are complex and the parents of couples in the PSID may have divorced, remarried, or widowed while they were growing up. Parental education in the PSID was asked using the question “How much education did your mother (father) complete?” thus leaving it up to the respondent to decide which individuals in their lives best fit these concepts. To the extent that family structure varies across the life course, the concept of social origins is measured with error, and our estimates of exchange will be attenuated. The fact that we find such strong evidence for exchange suggests that noisiness introduced by family complexity or other unmeasured factors are not so large that exchange cannot be detected.

### Categorization of Education and Social Origins

We measure social origins and education using a seven-category scheme. The categories are determined by relative frequency and do not correspond to any of the milestones of educational attainment. To test the robustness of our results to alternate schemes, we estimated the models using both a four-category education classification (<12, 12, 13–15, and ≥16 years of schooling) and a five-category classification (<9, 10–11, 12, 13–15, and ≥16 years of schooling) for spouses and parents. In both cases, our results were very similar to those presented here (see the online supplement).

### Treatment of Missing Data

Husbands and wives who did not report a parent’s education are excluded from the analysis. We tested the sensitivity of our results to the exclusion of these couples by setting the education of the parent with missing education values equal to that of the non-missing parent. This is equivalent to assuming that the non-missing parent’s education is the only parent whose education matters in marriage decisions or, alternatively, that both mother’s and father’s education matter but that these parents are all educationally homogamous. Again, our results are robust to the inclusion of these couples.

### Negative Binomial Models

We estimated our log-linear models as Poisson models. To account for overdispersion, we reestimated equivalent negative binomial models, as advocated by Rosenfeld (2005) (see the online supplement). Our results are not sensitive to the type of model used. We cannot reject the null hypothesis that the data are Poisson distributed in models 3–6 (using the Stata command estat gof). Thus, our finding that the results are similar across the two types of models is not surprising given that the negative binomial distribution is equivalent to a Poisson distribution when there is no overdispersion, as we find for models 3–6.

### Period Variation

Our analysis combines 55 years of marriage data. During this period, the educational attainment of men and women, in particular, increased, and patterns of educational homogamy and hypergamy have also changed (Schwartz and Mare 2005). We divided data into three periods—1968–1979, 1980–1991, and 1992–2013—and found robust evidence of exchange in each period. In addition, there are no period differences in the gender symmetric pattern of exchange between 1968–1979, 1980–1991, and 1992–2013.

## Discussion and Conclusion

Despite the romantic love ideal, social origins continue to play an important role in marriage formation in contemporary American society. Previous research has found that individuals tend to marry those of similar social origins above and beyond the tendency to match on their own educational attainment (Blau and Duncan 1967; Kalmijn 1991; Charles et al. 2013) and that parental education improves women’s chances of marrying highly educated men net of their own education (Blackwell 1998). Our study has additionally demonstrated that status exchange characterizes patterns of intermarriage. Among intermarried couples, the spouse with higher status on one trait tends to be the one with lower status on the other. The magnitude of exchange is not small. Our models indicate that exchange marriages are at least 34 percent more common than marriages in which either husbands or wives have the advantage on both education and social origins, controlling for the joint distribution of correlated traits, homogamy, and hypergamy.

Our conclusion is based on multiple tests designed to identify the pattern of exchange from confounding factors of correlated traits and general tendencies toward homogamy and hypergamy. Comparisons of various tests indicate that the single most important confounding factor is the within-person positive correlation between social origin and education—that is, the tendency for people who are highly educated to come from more privileged backgrounds than those with less education. Once we control for this, we find a persistent pattern of exchange. The specification of homogamy did not make a qualitative difference for our results.

Although we have no direct evidence on other types of exchange, our results strongly suggest that scholars should pay close attention to within-person positive correlations between traits of interest—for instance, the tendency for those with higher incomes to be more physically attractive in studies of the exchange of income for attractiveness or the association between race and education in studies of race–SES exchange (also see McClintock 2014). Controlling for these correlated traits led us to find robust evidence for exchange. Models that control for correlated traits are preferable to those that do not because we are interested in patterns of marital sorting that occur within a social context, with distributions of men’s and women’s traits already given a priori.

An objection could be raised that we did not control for traits or factors that are correlated with both education and social origin, such as occupational standing, income, and year of marriage. Are our results robust to omitted variable bias? If we had controlled for such variables, spousal similarities in social origin and education would likely be lower. However, our main result of status exchange would likely stand. This is because exchange is operationalized as a negative association between spousal differences in social origin and educational attainment, whereas most unmeasured variables that are correlated with both traits, such as wealth or cultural capital, would introduce a positive association between spouses in relative status. In other words, if there were omitted variable bias, in most cases we would expect it to work against the exchange hypothesis.

Status exchange has been tested elsewhere as a positive cross-trait correlation between spouses—e.g., the positive correlation between wives’ physical attractiveness and husbands’ socioeconomic status (Elder 1969; Taylor and Glenn 1976; Udry 1977). Such tests are prone to omitted variable bias, as unmeasured traits can easily provide an alternative explanation. Indeed, for the exchange of attractiveness and SES, two studies have shown that controlling for husband’s physical attractiveness eliminates the positive correlation between wife’s physical attractiveness and husband’s SES (Stevens, Owens, and Schaefer 1990; McClintock 2014). In contrast, our test of exchange as a negative correlation between spouses in relative statuses in two traits is more robust—not only because we have taken into account both spouses’ traits but also because unmeasured variables tend to make the test conservative.

Some scholars have claimed that marriage markets are not well characterized by status exchange but that individuals primarily seek to match on traits (e.g., Rosenfeld 2005; McClintock 2014). We find that homogamy is undoubtedly the primary marital sorting mechanism but there is also strong and robust evidence for exchange, at least with respect to social origin and education. Furthermore, our analysis of the pattern of exchange by couples’ relative endowment suggests that individuals exchange traits they have relatively more of for those that they have relatively less of. This result suggests that exchange marriage is not merely an auxiliary to homogamy—as an alternative way of achieving balance between partners—but rather that men and women utilize exchange marriage to adjust their resource portfolios for the betterment of themselves and potentially the next generation.

## Supplementary Material

Supplementary File

## Figures and Tables

**Table 1:** Classification of marriages by spouse’s relative education and social origin.

		Spouse’s Relative Social Origin		
		HO <WO (hypogamy)	HO = WO (homogamy)	HO >WO (hypergamy)
Spouse’s Relative Education	HE < WE (hypogamy)	[1,1]	[1,2]	[1,3]
		Wife-advantaged		Exchange of wife’s E for husband’s O
	HE = WE (homogamy)	[2,1]	[2,2]	[2,3]
	HE > WE (hypergamy)	[3,1]	[3,2]	[3,3]
		Exchange of husband’s E for wife’s O		Husband-advantaged

Notes: HE = husband’s education; WE = wife’s education; HO = husband’s social origin; WO = wife’s social origin; hypogamy = wives "marry down"; homogamy = spouses share traits; hypergamy = husbands "marry down." Cell row (i) and column (j) positions are shown in brackets [i, j].

**Table 2:** Percent distribution of marriages by spouse’s relative education and social origin.

		Spouse’s Relative Social Origin			
		HO < WO (hypogamy)	HO = WO (homogamy)	HO > WO (hypergamy)	row percent
Spouse’s Relative Education	HE < WE (hypogamy)	12.7	8.3	6.9	27.9
		[1,1]	[1,2]	[1,3]	
	HE = WE (homogamy)	16.7	14	14.4	45.1
		[2,1]	[2,2]	[2,3]	
	HE > WE (hypergamy)	8.5	8.2	10.4	28.1
		[3,1]	[3,2]	[3,3]	
	Column percent	37.8	30.5	31.7	100

Notes: HE = husband’s education; WE = wife’s education; HO = husband’s social origin; WO = wife’s social origin; hypogamy = wives "marry down"; homogamy = spouses share traits; hypergamy = husbands "marry down." Cell row (i) and column (j) positions are shown in brackets [i, j]. Total sample size n = 7,398.

**Table 3:** Simple and complex tests of status exchange.

	HE for WO	WE for HO
"Simple" Tests Using Tabular Analyses		
1. One-group test^a^	0.66†	0.66†
2. Two-group test controlling for hypergamy^a^	0.68†	0.65†
"Complex" Tests Using Log-Linear Models		
3. Two-group test controlling for hypergamy & intertrait correlations^a^	2.07†	2.1†
4. Two-group test controlling for hypergamy, intertrait correlations, & homogamy on education and social origins^b^		
Model 1: simple homogamy	2.38†	2.64†
	(0.20)	(0.23)
Model 2: variable homogamy	2.08†	2.29†
	(0.17)	(0.20)
Model 3: distance parameters	1.34†	1.41†
	(0.11)	(0.12)
Model 4: distance + variable homogamy	1.34†	1.42†
	(0.11)	(0.12)
Model 5: quasi-symmetry	1.34†	1.42†
	(0.11)	(0.12)
Model 6: saturated HE × WE and HO × WO	1.35†	1.42†
	(0.11)	(0.12)

Notes: HE = husband’s education; WE = wife’s education; HO = husband’s social origin; WO = wife’s social origin.

† *p* < 0.01;

* *p* < 0.05.

Numbers in parentheses are standard errors

a Significance levels for these tests are calculated using bootstrapped confidence intervals following Efron and Tibshirani (1994:168–73, 227). Each test is calculated by randomly selecting 1,000 samples of size n = 7,398 from the original sample with replacement.

b Model specifications are as follows. Model 1: simple homogamy distinguishes between homogamous matches (diagonals of the table) and heterogamous matches (off-diagonals only). Model 2: variable homogamy distinguishes between homogamous and heterogamous matches and allows the strength of homogamy to vary by the level of education or social origin. Model 3: distance parameters specify that the tendency to marry varies by the couple’s distance in status in education and social origin (|HO-WO| and |HE-WE|, respectively). Model 4: distance + variable homogamy is a hybrid model that contains parameters from both model 2 and model 3. Model 5: quasi-symmetry allows matching tendencies to be unique for each combination of couples’ statuses but constrains the parameters to be symmetric by sex. Model 6: saturated HE × WE and HO × WO saturates the two-way interactions between husbands’ and wives’ education and between husbands’ and wives’ social origins; it does not constrain the pattern to be symmetric by sex.

**Table 4:** Variation in exchange by spouses’ relative endowments of education and social origin.

	Type of Exchange
	HE for WO
(A1) Subsample HE > HO & WE ≤ WO	1.28*
	(0.16)
(A2) Other couples	1.07
	(0.12)
p-value (A1) = (A2)	0.205
	WE for HO
(B1) Subsample WE > WO & HE ≤ HO	1.63†
	(0.19)
(B2) Other couples	1.22
	(0.15)
p-value (B1) = (B2)	0.016*

Notes: HE = husband’s education; WE = wife’s education; HO = husband’s social origin; WO = wife’s social origin.

† *p* < 0.01;

* *p* < 0.05.

Numbers in parentheses are standard errors. Total sample size n = 7,398.

## Footnotes

1 The programs used to estimate the results are available on the first author’s website, and the individual-level data is available from the first author upon request.

2 These statistics mask considerable time variation in the likelihood that men and women “marry up” with respect to education. Between the 1970s and mid-1980s, women were more likely to “marry up” while those married since then were more likely to “marry down” (Schwartz and Mare 2005). We consider time variation in our results further below.

3 If the tendency for educational hypergamy is systematically different for origin-heterogamous couples and origin-homogamous couples, then estimates of exchange from this test may be biased.

4 The terms HE, HO, HE × HO control for the distribution of husbands in each category of education by social origin, while the terms WE, WO, WE × WO control for the distribution of wives. Note that we are not controlling for the characteristics of the general population; this test only controls for the characteristics of those who are married.

5 The ratio of Kalmijn’s hypergamy ratios for mixed-race and same-race couples is equivalent to our odds ratio of exchange marriage.

6 Interactions between dummies of relative advantage and homogamy (DHE = WE and DHO = WO), hypergamy (DHE > WE and DHO > WO), DHE = WE, HO > WO, and DHE = WE, HO < WO are also included in the model but the coefficients not presented.

7 The occupation categories are (1) laborers, service workers, farm laborers, farmers, and farm managers; (2) operatives and kindred workers; (3) craftsmen, foremen, and kindred workers; (4) armed services, protective, and other workers; (5) self-employed businessmen, clerical workers, and sales workers; 6) managers; 7) professionals and technical workers.
