# Supplementary material for: Marrying Up by Marrying Down: Status Exchange between Social Origin and Education in the United States
Source: Sociol Sci. Author manuscript; Available in PMC 2017 Nov 1. (PMC5214284; doi:10.15195/v3.a44)
Supplement: Supplementary File [file NIHMS835010-supplement-Supplementary_File.pdf]

sociological science

Supplement to:

Schwartz, Christine R., Zhen Zeng, and Yu Xie. 2016. "Marrying Up by Marrying Down: Status Exchange between Social Origin and Education in the United States." Sociological Science 3: 1003-1027.

Appendix Table 1: Goodness of Fit Statistics for Log-Linear Models of Exchange

	L^2	df	p	BIC	AIC
Model 1: simple homogamy	23669	104	0.000	7510	6784
Model 2: variable homogamy	24190	116	0.000	7096	6288
Model 3: distance parameters	25477	114	0.000	5791	4996
Model 4: distance + variable homogamy	25581	126	0.000	5794	4916
Model 5: quasi-symmetry	25611	144	0.000	5924	4922
Model 6: saturated HExWE and HOxWO	25642	172	0.000	6143	4947

Notes: HE = husband's education; WE = wife's education; HO = husband's social origin; WO = wife's social origin. Akaike's (1974) and Schwarz's (1978) Bayesian information criteria estimated using estat ic in Stata. n = 7,398.

Appendix Table 2: Tests of Status Exchange Using Alternative Specifications

	HE for WO	WE for HO
4-category education classification (<12, 12, 13-15, and ≥16)		
Two-group test controlling for hypergamy, inter-trait correlations, & homogamy on education and social origins		
Model 1: simple homogamy	2.22 ***	3.01 ***
Model 2: variable homogamy	1.79 ***	2.37 ***
Model 3: distance parameters	1.41 ***	1.87 ***
Model 4: distance + variable homogamy	1.42 ***	1.89 ***
Model 5: quasi-symmetry	1.42 ***	1.89 ***
Model 6: saturated HExWE and HOxWO	1.41 ***	1.90 ***
5-category education classification (<9, 10-11, 12, 13-15, and ≥16)		
Two-group test controlling for hypergamy, inter-trait correlations, & homogamy on education and social origins		
Model 1: simple homogamy	2.20 ***	2.86 ***
Model 2: variable homogamy	1.87 ***	2.30 ***
Model 3: distance parameters	1.31 ***	1.63 ***
Model 4: distance + variable homogamy	1.33 ***	1.65 ***
Model 5: quasi-symmetry	1.34 ***	1.65 ***
Model 6: saturated HExWE and HOxWO	1.34 ***	1.65 ***
Negative binomial models		
Two-group test controlling for hypergamy, inter-trait correlations, & homogamy on education and social origins		
Model 1: simple homogamy	2.53 ***	1.94 ***
Model 2: variable homogamy	2.46 ***	1.89 ***
Model 3: distance parameters	1.47 ***	1.36 **
Model 4: distance + variable homogamy	1.44 ***	1.38 **
Model 5: quasi-symmetry	1.44 ***	1.38 **
Model 6: saturated HExWE and HOxWO	1.43 ***	1.39 **

Notes: HE = husband's education; WE = wife's education; HO = husband's social origin; WO = wife's social origin. *p < .05; **p < .01; ***p < .001. *n* = 7,398.
